# Supplementary material for: Failures of nerve regeneration caused by aging or chronic denervation are rescued by restoring Schwann cell c-Jun
Source: eLife. 2021 Jan 21;10:e62232. doi: 10.7554/eLife.62232 (PMC7819709; doi:10.7554/eLife.62232)
Supplement: Figure 6—source data 1. [file elife-62232-fig6-data1.docx]

Figure 6

6A – Schwann cell numbers in injured young and aged nerves, 4d cut

| **Genotype** | **Age** | **Mean** | **SEM** | **n** | **Comparisons made** | **p** |
| --- | --- | --- | --- | --- | --- | --- |
| WT | Young | 201.8 | 21.39 | 4 |  | NS |
| OE/+ | Young | 163.8 | 15.77 | 4 |  | NS |
| WT | Old | 181.5 | 28.79 | 4 |  | NS |
| OE/+ | Old | 197.8 | 28.74 | 4 |  | NS |

Two-way ANOVA with Tukey’s test

6B– EM macrophage density in injured young and aged nerves, 4d cut

| **Genotype** | **Age** | **Mean** | **SEM** | **n** | **Comparisons made** | **p** |
| --- | --- | --- | --- | --- | --- | --- |
| WT | Young | 13.76 | 3.547 | 4 |  | NS |
| OE/+ | Young | 12.37 | 1.438 | 4 |  | NS |
| WT | Old | 7.198 | 0.5636 | 4 |  | NS |
| OE/+ | Old | 8.245 | 1.247 | 4 |  | NS |

Two-way ANOVA with Tukey’s test

6C– EM macrophage numbers in injured young and aged nerves, 4d cut

| **Genotype** | **Age** | **Mean** | **SEM** | **n** | **Comparisons made** | **p** |
| --- | --- | --- | --- | --- | --- | --- |
| WT | Young | 127.5 | 31.56 | 4 |  | NS |
| OE/+ | Young | 109.8 | 12.31 | 4 |  | NS |
| WT | Old | 171.3 | 40.76 | 4 |  | NS |
| OE/+ | Old | 138.5 | 10.90 | 4 |  | NS |

Two-way ANOVA with Tukey’s test

6D – EM fibroblast density in injured aged nerves, 4d cut

| **Genotype** | **Age** | **Mean** | **SEM** | **n** | **Comparisons made** | **p** |
| --- | --- | --- | --- | --- | --- | --- |
| WT | Young | 2.615 | 0.5446 | 4 |  | NS |
| OE/+ | Young | 3.885 | 0.8566 | 4 | Young OE/+ vs old WT and old OE/+ | Both  P=<0.05  * |
| WT | Old | 1.238 | 0.1460 | 4 |  |  |
| OE/+ | Old | 1.368 | 0.1145 | 4 |  |  |

Two-way ANOVA with Tukey’s test

6E – EM fibroblast numbers in injured aged nerves, 4d cut

| **Genotype** | **Age** | **Mean** | **SEM** | **n** | **Comparisons made** | **p** |
| --- | --- | --- | --- | --- | --- | --- |
| WT | Young | 24 | 4.062 | 4 |  | NS |
| OE/+ | Young | 34.00 | 7.071 | 4 |  | NS |
| WT | Old | 30.25 | 8.731 | 4 |  | NS |
| OE/+ | Old | 23.50 | 0.5000 | 4 |  | NS |

Two-way ANOVA with Tukey’s test

6F – EM nerve area in Age

| **Genotype** | **Age** | **Mean** | **SEM** | **n** | **Comparisons made** | **p** |
| --- | --- | --- | --- | --- | --- | --- |
| WT | Young | 93656 | 3948 | 4 | Young WT vs Old WT | p=<0.01  ** |
| OE/+ | Young | 88914 | 2368 | 4 |  |  |
| WT | Old | 232031 | 39117 | 4 | Young OE/+ vs Old WT | p=<0.01  ** |
| OE/+ | Old | 176766 | 19715 | 4 |  |  |

Two-way ANOVA with Tukey’s test

6G– EM Schwann cell counts in short and long term denervation

| **Genotype** | **Time** | **Mean** | **SEM** | **n** | **Comparisons made** | **p** |
| --- | --- | --- | --- | --- | --- | --- |
| WT | 2W | 257.3 | 13.21 | 7 | 2W WT vs 10W WT | P=<0.01  ** |
| OE/+ | 2W | 244.9 | 12.67 | 7 | 2W OE vs 10W OE and 10W WT | Both P=<0.05  * |
| WT | 10W | 182.0 | 8.959 | 6 | 10W WT vs 10W OE | P= < 0.0001  **** |
| OE/+ | 10W | 301.8 | 17.27 | 5 |  |  |

Two-way ANOVA with Tukey’s test

6H – EM macrophage density in short and long term denervation

| **Genotype** | **Time** | **Mean** | **SEM** | **n** | **Comparisons made** | **p** |
| --- | --- | --- | --- | --- | --- | --- |
| WT | 2W | 6.206 | 0.7784 | 7 |  | NS |
| OE/+ | 2W | 6.144 | 1.055 | 7 |  | NS |
| WT | 10W | 4.330 | 0.2989 | 5 |  | NS |
| OE/+ | 10W | 3.583 | 1.130 | 5 |  | NS |

Two-way ANOVA with Tukey’s test

6I– EM macrophage numbers in short and long term denervation

| **Genotype** | **Time** | **Mean** | **SEM** | **n** | **Comparisons made** | **p** |
| --- | --- | --- | --- | --- | --- | --- |
| WT | 2W | 80.00 | 9.016 | 7 | 2W WT vs 10W WT and 10W OE/+ | Both p=<0.01  ** |
| OE/+ | 2W | 83.00 | 11.91 | 7 |  |  |
| WT | 10W | 21.20 | 1.020 | 5 | 2W OE/+ vs 10W WT | P=<0.001  *** |
| OE/+ | 10W | 24.60 | 4.400 | 5 | 2W OE/+ vs 10W OE/+ | p=<0.01  ** |

Two-way ANOVA with Tukey’s test

6J – EM fibroblast density in short and long term denervation

| **Genotype** | **Time** | **Mean** | **SEM** | **n** | **Comparisons made** | **p** |
| --- | --- | --- | --- | --- | --- | --- |
| WT | 2W | 2.347 | 0.3229 | 7 | 2W WT vs 10W WT | p=<0.01  ** |
| OE/+ | 2W | 2.456 | 0.2889 | 7 | 2W OE/+ vs 10W WT | p=<0.01  ** |
| WT | 10W | 5.307 | 1.032 | 5 | 10W OE/+ vs 10W WT | P=<0.05  * |
| OE/+ | 10W | 2.639 | 0.6052 | 5 |  |  |

Two-way ANOVA with Tukey’s test

6K – EM fibroblast numbers in short and long term denervation

| **Genotype** | **Time** | **Mean** | **SEM** | **n** | **Comparisons made** | **p** |
| --- | --- | --- | --- | --- | --- | --- |
| WT | 2W | 30.86 | 4.543 | 7 |  | NS |
| OE/+ | 2W | 33.71 | 3.980 | 7 |  | NS |
| WT | 10W | 25.00 | 2.757 | 5 |  | NS |
| OE/+ | 10W | 19.00 | 3.464 | 5 |  | NS |

Two-way ANOVA with Tukey’s test

6L – EM nerve area short and long term denervation

| **Genotype** | **Time** | **Mean** | **SEM** | **n** | **Comparisons made** | **p** |
| --- | --- | --- | --- | --- | --- | --- |
| WT | 2W | 130262 | 5097 | 7 | 2W WT vs 10W WT and OE/+ | Both P= < 0.0001  **** |
| OE/+ | 2W | 138255 | 4782 | 7 | 2W OE/+ vs 10W WT and OE/+ | Both P= < 0.0001  **** |
| WT | 10W | 50321 | 5339 | 5 | 10W WT vs 10W OE/+ | P=<0.05  * |
| OE/+ | 10W | 77275 | 7520 | 5 |  |  |

Two-way ANOVA with Tukey’s test
